# Expression profiling of stem cell signaling alters with spheroid formation in CD133^high^/CD44^high^ prostate cancer stem cells

**DOI:** 10.3892/ol.2014.1992

**Published:** 2014-03-24

**Authors:** GULPERI OKTEM, AYHAN BILIR, RUCHAN USLU, SEVINC V. INAN, SIRIN B. DEMIRAY, HARIKA ATMACA, SULE AYLA, OGUN SERCAN, AYSEGUL UYSAL

**Affiliations:** 1 Department of Histology and Embryology, Faculty of Medicine, Ege University, Bornova, Izmir 35100, Turkey; 2 Department of Histology and Embryology, Istanbul Medical Faculty, Istanbul University, Capa, Istanbul 34098, Turkey; 3 Department of Medical Oncology, Faculty of Medicine, Ege University, Bornova, Izmir 35100, Turkey; 4 Department of Histology and Embryology, Faculty of Medicine, Manisa 45030, Turkey; 5 Department of Biology, Faculty of Science and Art, Celal Bayar University, Manisa 45030, Turkey; 6 Zeynep Kamil Gynecology and Maternity Training and Research Hospital, Istanbul 34668, Turkey; 7 Department of Medical Biology, Faculty of Medicine, Dokuz Eylul University, Bornova, Izmir 35340, Turkey

**Keywords:** cancer stem cell, stem cell-related genes, spheroid, prostate cancer

## Abstract

Cancer stem cells (CSC) isolated from multiple tumor types differentiate *in vivo* and *in vitro* when cultured in serum; however, the factors responsible for their differentiation have not yet been identified. The first aim of the present study was to identify CD133^high^/CD44^high^ DU145 prostate CSCs and compare their profiles with non-CSCs as bulk counterparts of the population. Subsequently, the two populations continued to be three-dimensional multicellular spheroids. Differentiation was then investigated with stem cell-related genomic characteristics. Polymerase chain reaction array analyses of cell cycle regulation, embryonic and mesenchymal cell lineage-related markers, and telomerase reverse transcriptase (*TERT*) and *Notch* signaling were performed. Immunohistochemistry of *CD117*, *Notch1*, *Jagged1*, *Delta1*, *Sox2*, *c-Myc*, *Oct4*, *KLF4*, *CD90* and *SSEA1* were determined in CSC and non-CSC monolayer and spheroid subcultures. Significant gene alterations were observed in the CD133^high^/CD44^high^ population when cultured as a monolayer and continued as spheroid. In this group, marked gene upregulation was determined in *collagen type 9 α1*, *Islet1* and *cyclin D2. Jagged1*, *Delta-like 3* and *Notch1* were respectively upregulated genes in the Notch signaling pathway. According to immunoreactivity, the staining density of *Jagged1*, *Sox2*, *Oct4* and *Klf-4* increased significantly in CSC spheroids. Isolated CSCs alter their cellular characterization over the course of time and exhibit a differentiation profile while maintaining their former surface antigens at a level of transcription or translation. The current study suggested that this differentiation process may be a mechanism responsible for the malignant process and tumor growth.

## Introduction

It has been widely accepted that tumor growth is sustained by a rare subpopulation of putative cancer stem cells (CSCs)/progenitor-like cells that share specific characteristics with normal stem cells, namely self-renewal, clonogenicity and multipotency (1–3). Previous investigations have shown that a number of tumors may actually arise from the transformation of progenitor cells rather than stem cells (4,5). Normal stem cells and CSCs share significant properties, such as heterogeneity and plasticity. Maturation and differentiation are important in cancer cell heterogeneity, and tumor cell heterogeneity may result from clonal evolution driven by genetic instability of stem-like cells, frequently called CSCs or tumor-initiating cells (6). Cells in this heterogeneous population exist in various stages throughout their lifetime. During early tumor development or in unperturbed tumor conditions, CSCs mainly undergo one-way maturation by developing into tumor progenitor cells and even differentiated tumor cells (7). It is possible to assume that these differentiated cells may arise from CSCs, which have self-renewal capacity and/or phenotypically differentiated tumor cells that functionally possess low or no tumor-regenerating capacity (non-CSCs/bulk population). CSCs are the cell subpopulation that are most likely responsible for treatment failure and cancer recurrence, while the bulk population of tumor cells exhibit low self-renewal capacity and a higher probability of terminal differentiation (i.e. transit-amplifying cancer progenitor cells) (8).

CSCs have been previously isolated and identified using common cell surface markers in the majority of cancer types, including brain (9,10), kidney (11), liver (12,13), colon (14), pancreas (15) and prostate (16). CD133, also known as prominin-1 or *AC133* (a glycoprotein comprising of five transmembrane domains), has been described as a marker of stem cells in several organs and appears to be the CSC marker for a number of tumor types (17). However, there have been accumulating results demonstrating that CD133^+^ and CD133^−^ subpopulations are tumorigenic in metastatic glioblastoma and colon cancer (18–20).

CD44 is a member of the cell adhesion protein family and the expression of several CD44 proteins has been found to correlate with aggressive stages of various types of human cancer (21). An evident function of the CD44 family members is their alternative splicing. Previously, Ponta *et al* demonstrated that CD44 family members differ in the extracellular domain by the insertion of variable regions through alternative splicing (22). A small subset of CD44^+^ cells in prostate cell cultures and xenograft tumors are more tumorigenic, proliferative, clonogenic and metastatic as compared with the CD44^−^ subpopulation. This CD44^+^ subset expresses higher mRNA levels of several genes that are characteristic of embryonic stem cells (23). In addition, Collins *et al* have shown that prostate cancer tumorigenic cells have a CD44^+^/1α2β1^high^/CD133^+^ phenotype (24).

A challenge has been encountered with regard to the enrichment of CSCs from the established cell lines of a variety of solid tumors that develop as three-dimensional (3D) cell cultures. The 3D spheroid model is a new technique for the propagation of cells *in vitro* using serum-free medium and cultured under low-adherence conditions (25). An additional usage of spheroids constitutes the liquid overlay technique, namely multicellular tumor spheroids (26)

The 3D spheroid model presents a convenient model to investigate cancer cells and has been increasingly used for this purpose. It reproduces *in vitro* results in accordance with *in vivo* results and generates significant *in vitro* characteristics not observed in monolayers or suspension cultures.

The present study hypothesized that the structure of CSCs may show differentiation when compared with non-CSCs, and differentiation of stem cell markers may aid therapeutic strategies of cancer. Therefore, the current study describes approaches to present and analyze the differentiation properties of human prostate CSCs within 3D spheroids, which may serve as the basis for defining the gene and protein trace of CSCs.

## Materials and methods

### Cell culture conditions and reagents

The DU145 human prostate cancer cell line was supplied by the American Type Culture Collection (Manassas, VA, USA) and was grown in monolayer culture in Dulbecco’s modified Eagle’s medium-F12 (DMEM-F12; Biological Industries Israel Beit-Haemek Ltd., Kibbutz Beit-Haemek, Israel) supplemented with 10% heat-inactivated fetal calf serum (Gibco, Invitrogen Life Science, Paisley, UK), 100 U/ml penicillin and 100 μg/ml streptomycin (Sigma-Aldrich, St Louis, MO, USA). Cells in semi-confluent flasks were harvested using 0.05% trypsin (Sigma-Aldrich), centrifuged (Nüve NF200, Laboratory and Sterilization Technology, Ankara, Turkey) following the addition of DMEM-F12 for trypsin inactivation and then resuspended in culture medium. The antibodies used consisted of *C-kit* (sc-168), *Notch1* (sc-6014), *Jagged1* (sc-6011) and *Delta1* (sc-8155) (all 1:100; Santa Cruz Biotechnology, Inc., Santa Cruz, CA, USA), *Sox2* (1:300; Abnova, Taipei, Taiwan), *c-Myc* (1:300; Santa Cruz Biotechnology, Inc.), *Oct4* and *Klf4* (1:300, Abcam, Cambridge UK), *CD90* (THY-1; 1:300, Abcam) and *SSEA1* (1:300, Abcam), secondary antibody (sc-2053; Histostain^®^-Plus Streptavidin-Peroxidase; Gibco, Invitrogen Life Technologies and Santa Cruz Biotechnology, Inc.).

### Fluorescence-activated cell sorting (FACS) and experimental groups

For FACS (Facs Aria, BD Biosciences, San Jose, CA, USA), cells were detached using non-enzymatic cell dissociation solution (Sigma-Aldrich) and ~5×10^4^ cells were incubated with antibody [dilution of 1:100 in FACS wash (0.5% bovine serum albumin, 2 mM NaN_3_ and 5 mM EDTA)] for 15 min at 4°C. An isotype and concentration-matched PE-labeled control antibody (Miltenyi Biotech, Bisley, UK) was used and the samples were labeled with PE-labeled CD133/1 (clone AC133/1; Miltenyi Biotech) and FITC-labeled CD44 (clone G44-26; BD Pharmingen, San Diego, USA). After 3–5 min, the cells were washed with FACS wash and resuspended. The cells were sorted into CD133^high^/CD44^high^ (CSC) and non-CSC subpopulations. The two subpopulations were cultured in two different settings, monolayer 2D culture or 3D multicellular tumor spheroid. Briefly, the experimental groups comprised of monolayer CSC (M^+^) and non-CSC (M^−^) and spheroid CSC (S^+^) and non-CSC (S^−^) subpopulations.

### Constitution of spheroids and sphere formation assay

For spheroid cultures, the tumor cells grown as monolayer were resuspended with trypsin and the clonogenic potential of various phenotypic populations was analyzed in a 3D non-adherent culture condition (plates coated with 3% Noble agar; Difco Laboaratories Inc., BD Diagnostic Systems, Detroit, MI, USA). The cells were counted, resuspended and plated with 1×10^3^ cells per well in a six-well plate. Two weeks following initiation, the plates were inspected for colony (sphere) growth. The number of colonies within each well was counted under the microscope (Olympus BX-51, Olympus, Hamburg, Germany) and images of representative fields were captured. First passage floating spheres were removed and gently disaggregated with a new 3% Noble agar-coated well.

### Polymerase chain reaction (PCR) array assay

Total RNA was extracted from CSCs and non-CSCs (miRNeasy kit; Qiagen, Hilden, Germany) and synthesis of cDNA was performed using the SuperArray kit (C-03; SABiosciences, Frederick, MD, USA). Stem cell-specific gene expression profiles were studied with the PCR array assay (SABiosciences) according to the manufacturer’s instructions. Briefly, total RNA was isolated from monolayer cell populations or whole floating spheroids. In total, ≤1 μg of total RNA was treated with DNase and cDNA was prepared using the RT^2^ first-strand kit. For each analysis, pairs of the test and control cDNA samples were mixed with RT^2^ qPCR master mix and distributed across the 96-well plate of the PCR array, each of which contained 84 stem cell-related probes and control housekeeping genes. After cycling with qPCR (LightCycler 480; Roche Diagnostics GmbH, Mannheim, Germany), the obtained amplification data (fold-changes in Ct values of all the genes) were analyzed using SABiosciences software and ≥1.5 fold-change was used for filtering criteria. Detailed analyses of telomerase reverse transcriptase (*TERT*); cyclin A2; cyclin D1 (*CCND1*); *CCND2*; cyclin E1 (*CCNE1*); cyclin-dependent kinase 1 (*CDK1*); GTP-binding protein (*CDC42*); E1A-binding protein (*EP300*); myelocytomatosis viral oncogene homolog (*MYC*); retinoblastoma 1; forkhead box A2 (*FOXA2*); actin, α, cardiac muscle 1 (*ACTC1*); achaete-scute complex homolog 2 (*ASCL2*); ISL LIM homeobox 1 (*ISL1*); keratin15 (*KRT15*); msh homebox 1 (*MSX1*); myogenic differentiation 1 (*MYOD1*); T, brachyury homolog (*T*); *NOTCH1; NOTCH2*; jagged1 (*JAG1*); Delta-like 1 (*DLL1*); *DLL3*; deltex homolog 1 (*DTX1*); *DTX2*; dishevelled, dsh homolog 1 (*DVL1*); K (lysine) acetyltransferase 2A (*KAT2A*); histone deacetylase 2 (*HDAC2*); numb homolog (*NUMB*); sex determining region Y, box 1 (*SOX1*); SOX2; aggrecan (*ACAN*); alkaline phosphatase, intestinal (*ALPI*); bone γ-carboxyglutamate protein (*BGLAP*); collagen, type I, α 1 (*COL1A1*); *COL2A1; COL9A1*; and peroxisome proliferator-activated receptor γ (*PPARG*) were performed.

### Immunohistochemical analysis

Immunohistochemistry was adapted and modified from our previous protocols (27). Briefly, monolayer cells were maintained in 24-well plates and fixed with paraformaldehyde. The spheroids were processed in routine histological processing for embedding in paraffin wax. Cells were incubated with primary antibodies overnight at 40°C in a humidity chamber. The modified Streptavidin-Peroxidase technique was then used. Following incubation with 3,3′-diaminobenzidine (Invitrogen Life Technologies), sections were counterstained with Mayer’s hematoxylin (Sigma-Aldrich). Immunoreactivity of molecules was assessed by light microscopy using Olympus BX-51 and C-5050 digital cameras. Staining was graded independently by two observers, blinded to the groups, who evaluated semi-quantitatively using the following scale: Mild, +; moderate, ++; and strong, +++.

## Results

### CD133^high^/CD44 ^high^ CSC and non-CSC subpopulation purity and sorting rates

Prior to performing the microarray, the purity of CSC and non-CSC samples was tested with CD133 and CD44. CSC sorting is performed 300 times per year in our laboratory (Molecular Embyology Laboratory, Department of Histology and Embryology, Faculty of Medicine, Ege University, Bornova, Turkey). Sorting rate analysis and purity of cells were evaluated sequentially and statistical analysis was performed using SPSS 15 (SPSS, Inc., Chicago, IL, USA). Rates were 9.67±5.4% for CSCs and 90.33±5.4 for non-CSCs. In order to confirm the flow cytometry analysis, cells were re-evaluated following sorting, and this analysis was repeated after one passage. Results showed that the purity of the cells was 85% and immunofluorescence staining yielded a cell purity of >85% in all samples.

### Analysis of TERT and cell cycle regulation gene products

**Figure 1:**
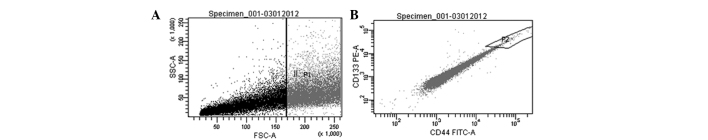
(A) Prostate CSCs sorted by FACSAria. (B) CD133^high^/CD44^high^ population (CSCs). Aside from this population, the remaining cells were classified as non-CSCs. CSCs, cancer stem cells.

**Figure 2:**
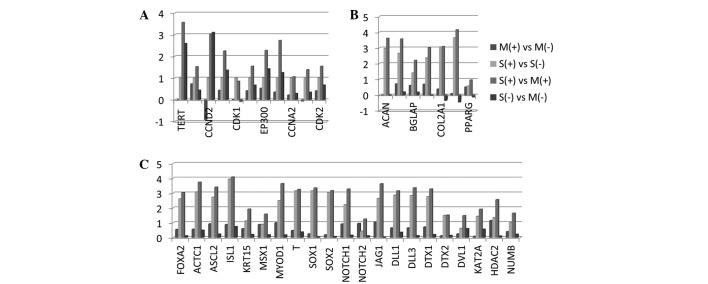
Microarray analysis in DU145 human prostate cell line in monolayer cells (M) and 3D spheroids (S), as well as in CD133^high^/CD44^high^ CSCs (S^+^ and M^+^) and their bulk counterpart non-CSCs (S^−^ and M^−^) cells was performed. (A) TERT and cell cycle regulation, (B) embryonic cell lineage and Notch signaling and (C) mesenchymal cell linage-related genes were demonstrated. These microarrays were analyzed to calculate the log-ratios. CSCs, cancer stem cells; TERT, telomerase reverse transcriptase; CCND2, cyclin D2; CDK1, cyclin-dependent kinase 1; EP300, E1A-binding protein; CCNA2, cyclin A2; CDK2, cyclin-dependent kinase 2; ACAN, aggrecan; BGLAP, bone γ-carboxyglutamate protein; COOL2A1, collagen, type II, α 1; PPARG, peroxisome proliferator-activated receptor γ; FOXA2, forkhead box A2; ACTC1, actin, α, cardiac muscle 1; ASCL2, achaete-scute complex homolog 2; ISL1, ISL LIM homeobox 1; KRT15, keratin15; MSX1, msh homebox 1; MYOD1, myogenic differentiation 1; T, T, brachyury homolog; SOX1/2, sex determining region Y, box 1/2; JAG1, jagged1; DLL1/3, Delta-like 1/3; DTX1/2, deltex homolog 1/2; DVL1, dishevelled, dsh homolog 1; KAT2A, K(lysine) acetyltransferase 2A; HDAC2, histone deacetylase 2; NUMB, numb homolog.

Following cell separation with FACS (Fig. 1), the differentially expressed genes of the DU145 human prostate cell line were analyzed in CD133^high^/CD44^high^ (CSCs) and their bulk counterpart (non-CSCs) cultured as monolayer cells or 3D spheroids. In general, notable differences were observed between the CSCs spheroid (S^+^) and monolayer (M^+^) groups. These two groups constituted of CSCs and TERT expression was increased significantly in the S^+^ group when compared with the M^+^ group (Fig. 2A). An additional large population of genes related to cell cycle regulation were analyzed, including *CCND1*, *CCND2*, *CCNE1, CDK1, CDC42, EP300* and *MYC*. These genes were upregulated in the S^+^ group versus the M^+^ group. CCND2 expression increased in the non-CSC counterpart of the S^−^ group compared with the S^+^ group. On the other hand, CCND2 was significantly reduced in the M^+^ group compared with the M^−^ group (Fig. 2A).

### Analysis of embryonic cell lineage and Notch signaling gene products

The differentiation of the embryonic cell linage genes of prostate CSC-enriched CD133^high^/CD44 ^high^ cells were determined and compared with the bulk counterparts in the monolayer and spheroid subpopulations. With respect to the embryonic cell lineages, *FOXA2, ACTC1, ASCL2, ISL1, KRT15, MSX1, MYOD1, T, SOX1* and *SOX2*, expression profiles were investigated. These genes were commonly upregulated in the CSC spheroid versus CSC monolayer cultures (S^+^ vs. M^+^], with significantly higher levels of *ISL1, ACTC1, MYOD1, ASCL2, SOX1, T* and *SOX2*. These genes were expressed at extremely low levels in the non-CSCs. The lowest expression was observed when comparing the S^−^ group with the M^−^ group.

The Notch signaling pathway is important in cell-to-cell communications that regulate multiple cell differentiation processes during embryonic and adult life (28). In the present study, the expression of *NOTCH1, NOTCH2, JAG1, DLL1, DLL3, DTX1, DTX2, DVL1, KAT2A, HDAC2* and *NUMB* was investigated. The expression of these genes significantly increased in the S^+^ versus the M^+^ populations compared with the other groups. The Notch signaling genes, *JAG1*, *DLL3, NOTCH1*, *DTX1* and *DLL1*, were of higher levels when compared with other upregulated genes (Fig. 2B).

### Analysis of mesenchymal cell linage gene products

The *ACAN, ALPI, BGLAP, COL1A1, COL2A1, COL9A1* and *PPARG* genes were evaluated. Significantly, the *COL9A1* gene was upregulated in the CSC spheroid as compared with the CSC monolayer. The expression of *COL2A1* and *COL9A1* genes was reduced in the non-CSC spheroids when compared with the monolayers (S^−^ vs. M^−^) (Fig. 2C).

### Immunohistochemical analysis of stem cell markers

**Figure 3:**
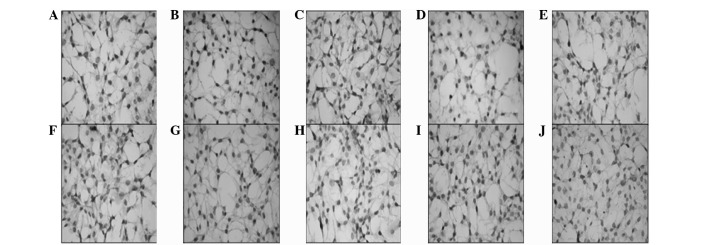
Immunohistochemistry of (A) CD117, (B) Notch1, (C) Jagged1, (D) Delta1, (E) Sox2, (F) c-Myc, (G) Oct4, (H) KLF4, (I) CD90 and (J) SSEA1 was determined in monolayer CSCs. Increased nucleus/cytoplasm ratio, decreased cell diameter and enhanced immunoreactivity were observed in the CD133^high^/CD44^high^ population. Although positive immunoreactivity was determined in all cells in all groups, the staining density of Jagged1, Sox2, Oct4 and Klf-4 increased significantly in the CD133^high^/CD44^high^ CSC population. CSCs, cancer stem cells.

**Figure 4:**
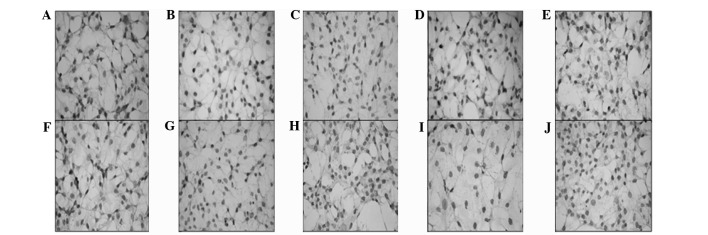
Immunohistochemistry of (A) CD117, (B) Notch1, (C) Jagged1, (D) Delta1, (E) Sox2, (F) c-Myc, (G) Oct4, (H) KLF4, (I) CD90 and (J) SSEA1 was determined in monolayer non-CSCs. Decreased nucleus/cytoplasm ratio, increased diameter and positive immunoreactivity was observed in the cells; however, the staining density of this non-CSC population was significantly decreased compared with the monolayer non-CSCs. CSCs, cancer stem cells.

**Figure 5:**
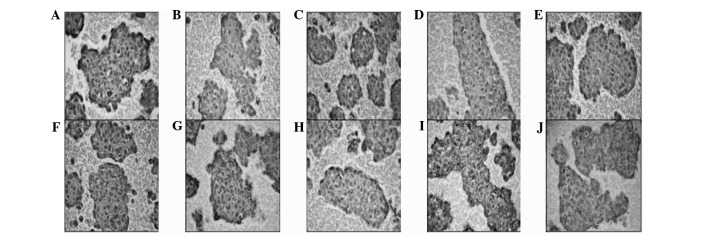
Immunohistochemistry of (A) CD117, (B) Notch1, (C) Jagged1, (D) Delta1, (E) Sox2, (F) c-Myc, (G) Oct4, (H) KLF4, (I) CD90 and (J) SSEA1 was determined in CSC spheroids. Marked immunoreactivity was observed in all groups. CSC, cancer stem cells.

**Figure 6:**
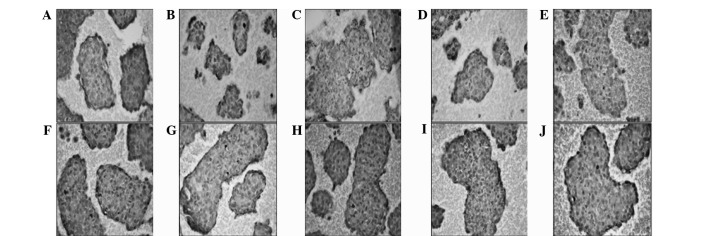
Immunohistochemistry of (A) CD117, (B) Notch1, (C) Jagged1, (D) Delta1, (E) Sox2, (F) c-Myc, (G) Oct4, (H) KLF4, (I) CD90 and (J) SSEA was determined in non-CSC spheroids. Notch1, Jagged1 and Delta1 immunoreactivity was moderate compared with the marked immunoreactivity of the other groups. CSC, cancer stem cell.

Results of the immunohistochemical analyses revealed that embryonic stem cell markers increased following the differentiation of CSCs when the cells constituted a spheroid formation. Immunohistochemistry of *CD117*, *Notch1*, *Jagged1*, *Delta1*, *Sox2*, *c-Myc*, *Oct4*, *KLF4*, *CD90* and *SSEA1* was determined in the various groups. Positive immunoreactivity was observed in CSCs and non-CSCs whether the cells were maintained in monolayer culture or as spheroid. The monolayer CSCs showed low (+) immunoreactivity scores (Fig. 3), while the monolayer non-CSCs (Fig. 4) showed moderate (++) immunoreactivity. Increased nucleus/cytoplasm ratios, decreased cell diameter and enhanced immunoreactivity were observed in the CD133^high^/CD44 ^high^ population. The staining density of *Jagged1*, *Sox2*, *Oct4* and *Klf-4* increased significantly in this monolayer CSCs population. On the other hand, strong (+++) immunoreactivity was observed in the CSC spheroids (Fig. 5) when compared with the non-CSC spheroids (Fig. 6). Among these spheroids, a moderate (++) immunoreactivity score was observed for *Notch1*, *Jagged1* and *Delta1* in the non-CSCs spheroids while strong (+++) immunoreactivity was observed in the other groups. Moreover, the highest immunoreactivity was observed in the CSC spheroid group when compared with the monolayer CSCs, monolayer non-CSCs or spheroid non-CSCs group.

## Discussion

Despite limited data in the previous literature, the differentiation of CSCs may be investigated. Cancer cells capable of undergoing proliferation have the ability to self-renew and their differentiation properties are unique to CSCs. In the present study, this differentiation hypothesis was examined by using an *in vitro* 3D-tumor differentiation spheroid model. The cells were found to alter their gene expression profiles during this process. Our hypothesis was supported by the observation that significant gene alterations were observed in the CD133^high^/CD44 ^high^ population when the monolayer cells were allowed to grow as spheroid. In this group, a marked upregulation was determined in *COL9A1* and *ISL1* compared with other genes. Type IX collagen is covalently bound to the surface of type II collagen fibrils within the cartilage extracellular matrix (29). Collagen IX is required for the integrity of collagen II fibrils and the regulation of vascular plexus formation (30). Additionally, Piotrowski *et al* previously demonstrated that complete loss of methylation affected *COL9A1* in tumors (31). However, no previous literature is available with regard to the role of *COL9A1* in cancer. In the current study, it was possible to assume that the upregulation of *COL9A1* correlates with the arrangement of the extrafibrillar proteoglycan, glycoprotein matrix and vascular development. *ISL1* is a LIM-homeodomain transcription factor that marks cell population and establishes endothelial, myocardial and smooth muscle cells. Previously, Schmitt *et al* demonstrated that *ISL1* is a reliable marker of pancreatic endocrine tumors and metastases (32). The present study reported, for the first time, that *ISL1* is an additional significantly upregulated gene in prostate spheroid CSCs. *ISL1*^+^ multipotent precursors have the potential of self-renewal and differentiation into endothelial, cardiomyocyte and smooth muscle lineages. These features highlight postnatal angiogenesis and vasculogenesis by improving the angiogenic properties of endothelial cells and mesenchymal stem cells (33). Angiogenesis is critical for tumor growth, and the *VEGF* pathway and *Notch* signaling are perhaps two of the most important mechanisms in the regulation of embryonic vascular development and tumor angiogenesis (34). According to our recent study, *Notch* signaling affects ovarian carcinomas and *Notch1* expression correlates with metastasis, while *Jagged1* expression correlates with tumor grade (27). However, it was demonstrated that in spheroids, all genes in Notch signaling are significantly upregulated, particularly *Jagged1*, *DLL3* and *Notch1*. High *Jagged1* expression has been demonstrated to predict a worse outcome in breast cancer (35,36), renal cell carcinoma (37) and colon adenocarcinoma (38). It has also been reported that high *Jagged1* expression is associated with prostate cancer recurrence (39). Furthermore, *Jagged1* signaling regulates hemangioma stem cell-to-pericyte/vascular smooth muscle cell differentiation (40). The abovementioned observations indicate that cellular organizations in CSCs accompany vascular development or extracellular structuring with the possible tendency of epithelial mesenchymal transition. The most upregulated cyclin was *CCND2*, which is implicated in cell differentiation and malignant transformation and is inactivated by promoter hypermethylation in several types of human cancer. High DNA methylation levels of *CCND2* cause deregulation of the G1/S checkpoint and correlate with clinicopathological features of tumor aggressiveness in breast and types of prostate cancer (41,42).

In conclusion, isolated CSCs in human tumors may alter their cellular characterization with time and exhibit differentiation by maintaining their former surface antigens at the level of transcription or translation. This differentiation may be a principal mechanism that is responsible for the malignant process and tumor growth. As demonstrated in the current study, upregulated genes of angiogenesis and mesenchymal transition or cellular tendency to the vascular development appear to be due to malignancy and tumor progression. Overall, these determinations indicated the differentiation of CSCs, but must be further validated with a series of patient samples derived from primary and/or metastatic lesions of prostate cancer.
